# Enhancing remediation by focusing on affective experience

**DOI:** 10.1111/medu.14670

**Published:** 2021-10-05

**Authors:** Valentina Colonnello

**Affiliations:** ^1^ Department of Experimental, Diagnostic and Specialty Medicine University of Bologna Bologna Italy

## Abstract

Colonnello offers the affective neuroscience view as a means for better understanding the role of emotions in remediation and nurturing synergistic interactions between research and education.

Mills and colleagues' recent review^1^ highlights how little attention emotions receive in remediation programmes, ‘which may be indicative of the persistence of a prior model that views emotion and cognition as discrete domains, with learning falling under the realm of cognition’. In doing so, it helps us determine important goals for medical education—one of which should be boosting research on emotions by applying knowledge from different fields. The authors also point out ‘potential areas for intervention, including emphasis on supportive remediation coaching relationships and greater attention to possible positive emotions’. Thus, their work helps identify several key issues that deserve to be the focus of future research studies on emotions in remediation and, more broadly, in medical education. This essay takes a brief look at some basic interrelated issues worth considering when designing those future studies: understanding emotions and learning within emotion theory perspectives, measuring emotions in remediation and using research findings in education.

With respect to the first issue, understanding emotions and learning, I would argue that it is important to broaden the current research scope in medical education to consider the more recent neuroevolutionary affective neuroscience perspective. This perspective offers an integrative framework for emotion theories and accepts the coexistence of various levels of emotion analysis that may be useful for researching emotions in remediation.^2^, ^3^ While Panksepp's affective neuroscience perspective has robustly informed recent clinical and basic sciences, it has received less attention in medical education. His model presents the interplay between emotion and cognition as being based on an evolutionary stratification of mental functions in which primary instinctual, action‐based, emotional processes are foundational for secondary learning processes and tertiary, cognitively mediated, thought. In humans, all the processes work in a hierarchical circular and causative fashion. A deeper understanding of learning processes and cognitively enriched emotions may, thus, be envisioned as being rooted in evolutionarily grounded basic emotional systems; these networks of interacting circuits are, in turn, refined by emotionally significant experiences and social context.

I propose broadening current research scopes in medical education to consider the more recent neuroevolutionary affective neuroscience perspective.

Learning processes and cognitively enriched emotions may, thus, be envisioned as being rooted in evolutionarily grounded basic emotional systems.

While a full description of the model is beyond the scope of this commentary (see Panksepp and Biven^2^ for such), neuroevolutionary affective neuroscience studies may well open a window to the emotional processes and behaviours underlying both the self‐conscious emotions that occur during remediation and the efficacy of remediation programmes. For example, self‐conscious cognitively enriched emotions such as shame and fear of stigma may see the involvement of the PANIC/separation distress emotional system; different degrees of activation of this system may lead to social support and reassurance seeking or to ‘surrender’, ‘give up’ feelings and reduced trust. Similarly, the two‐way route between emotions and learning is likely related to the involvement of the dopamine energised exploratory/SEEKING emotional system that fuels motivation and sustains all the other emotional systems.^3^, ^4^ Given that insight and motivation play a key role in the efficacy of remediation programmes,^5^ and the SEEKING system impacts learning by modulating attentive processes,^2^, ^3^ effective remediation programmes may be those that harness the power of exploratory/SEEKING, CARE and social PLAY emotional processes by both increasing feelings of social comfort and self‐confidence and nurturing insights and motivation. Whether these speculative hypotheses prove correct is immaterial for the moment as the general point is simply that the affective neuroscience perspective can help in structuring research studies that explore the emotional dynamics that may facilitate the development of supportive remediation relationships.

To move in that direction, careful attention must be paid to a second issue, the measurement of emotions. When choosing a measurement strategy, it is important to remember that emotions in remediation are experienced at different levels, not only through the cognitively mediated reasoning about one's own emotions that are most likely to be detected with self‐reports. Rather, there is value in adopting the straightforward strategy of designing research protocols that encompass a multimethod approach, including measures of cognitively mediated propositional descriptions of one's own emotions and action‐based expressions of emotions in daily context, such as those gathered by observing learners' moment‐to‐moment interactions with peers and supervisors and by measuring physiological activations. Analysis of non‐verbal behaviour and prosodic aspects of communication may be integrated with observations that tackle emotion‐based learned habits, attitudes and implicit bias, all of which are easily measurable in experimental tasks. By extending previous research into the effects care schema activation have on social cognition and attention to learning material,^6^, ^7^ researchers could investigate the impact of experimental manipulations on learners' emotional experiences toward remediation.

Emotions in remediation are experienced at different levels, not only through the cognitively mediated reasoning about one's own emotions.

Importantly, emotions in remediation would be better explored during early assessment experiences and using longitudinal studies, that is, well before remediation. Though remediation is an emblematic experience during medical education, it is likely preceded by experiences of frustration from unexpected feedback, fear of failure, anticipated or experienced shame and enthusiastic expectations regarding ‘second‐chance’ opportunities for learning or for improving social interactions with peers or supervisors. To this end, longitudinal research studies could focus on the expression of various emotions during regular coursework, assessments and on relational and contextual factors that may influence one's feelings toward remediation. Such longitudinal studies could aid in identifying and predicting the emotions related to remediation and the degree of individual variability in how remediation is expected, experienced and elaborated throughout the curriculum. They could also aid in identifying trajectories of emotional experience in remediation and who may need support to experience remediation as a positive turning point in their educational path.

Emotions in remediation would be better explored during early assessment experiences and using longitudinal studies.

Finally, a key issue in enhancing the link between research and learning is how best to harness research studies in educational contexts. As already stated elsewhere,^8^ and resonating with Balmer and colleagues' view,^9^ potentiating emotion research and promoting learners' psychological reasoning in medical education may be envisioned as a two‐way process that begins early in the medical education curriculum and involves both research on medical students' emotional processes and discussing those findings with the students. Their reflections and feedback in turn serve as a compass for new research questions and experiments.

Emotion research and promoting learners' psychological reasoning in medical education may be envisioned as a two‐way process.

Self‐emotional insights on one's own and others' emotionally driven behaviours and beliefs more likely occur when the reflection is anchored to concrete, emotionally self‐relevant, action‐triggering experiences. Thus, like the several emotionally salient challenges occurring early on during the curriculum, remediation represents a key opportunity for studying learners' emotions in context and for prompting learners to observe, with curiosity, their own and their peers' psychological processes, from emotions in actions to cognitively mediated beliefs to personal roles in affecting interpersonal interactions. Thus, we believe a research programme combined with educational attention to emotions in remediation holds great potential to yield insights that can be translated into testable interventions that support learners beyond the remediation experience itself, during the challenging times of continuing education and daily clinical work.
